# New Insights into 5hmC DNA Modification: Generation, Distribution and Function

**DOI:** 10.3389/fgene.2017.00100

**Published:** 2017-07-19

**Authors:** Dong-Qiao Shi, Iftikhar Ali, Jun Tang, Wei-Cai Yang

**Affiliations:** State Key Laboratory of Molecular Developmental Biology, National Center for Plant Gene Research (Beijing), Institute of Genetics and Developmental Biology, Chinese Academy of Sciences, University of Chinese Academy of Sciences Beijing, China

**Keywords:** 5-hydroxymethylcytosine, DNA hydroxylation, DNA demethylation, TET proteins, epigenetics

## Abstract

Dynamic DNA modifications, such as methylation/demethylation on cytosine, are major epigenetic mechanisms to modulate gene expression in both eukaryotes and prokaryotes. In addition to the common methylation on the 5th position of the pyrimidine ring of cytosine (5mC), other types of modifications at the same position, such as 5-hydroxymethyl (5hmC), 5-formyl (5fC), and 5-carboxyl (5caC), are also important. Recently, 5hmC, a product of 5mC demethylation by the Ten-Eleven Translocation family proteins, was shown to regulate many cellular and developmental processes, including the pluripotency of embryonic stem cells, neuron development, and tumorigenesis in mammals. Here, we review recent advances on the generation, distribution, and function of 5hmC modification in mammals and discuss its potential roles in plants.

## Introduction

**FIGURE 1:**
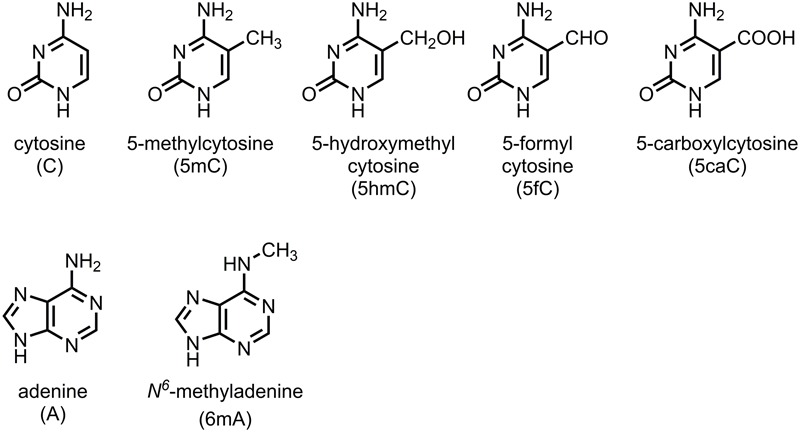
Different types of DNA modification.

From two decades of studies, it is clear that the primary sequence information of DNA can be enhanced by epigenetic modifications. There can be various epigenomes in different cell types, although there is only one genome of an organism. Since the discovery of the first modified base, 5-methylcytosine (5mC, methylation of cytosine at the 5-carbon position) in calf thymus DNA by Hotchkiss (1948), many studies showed that DNA methylation regulates gene expression. As the most common DNA modification in eukaryotes, 5mC is sometimes regarded as the fifth genetic code, in addition to the canonical “A, G, C, T” in DNA. Of the approximately 28 million CpG sites present in the human genome, 60–80% of the cytosines are methylated as 5mC (Smith and Meissner, 2013). In angiosperms, the genome-wide methylation level (GML) is as high as 43% in *Beta vulgaris* (with mean of 16%) (Alonso et al., 2015; Niederhuth et al., 2016; Vidalis et al., 2016). Increasing data suggest that the epigenetic information of the epigenome is maintained and translated by the dynamic activity of DNA methylases (writers) and demethylases (erasers) and reader proteins who recognize and interpret the information in both mammals and plants (He X.J. et al., 2011; Torres and Fujimori, 2015; Feinberg et al., 2016). However, in some organisms, such as yeast, *Caenorhabditis elegans, Drosophila melanogaster* and many other invertebrates, either no or only trace amounts of methylated cytosine are found (Wenzel et al., 2011; Capuano et al., 2014). Other DNA modifications, such as 5-hydroxymethylcytosine (5hmC), 5-formylcytosine (5fC), 5-carboxylcytosine (5caC), and *N*^6^-methyladenine (6mA) are found to play critical roles in many biological processes (Wion and Casadesus, 2006; Kriaucionis and Heintz, 2009; Tahiliani et al., 2009; He Y.F. et al., 2011; Ito et al., 2011; Pfaffeneder et al., 2011; Fu and He, 2012; Liu et al., 2016) (**Figure 1**).

The methyl moiety of 5mC can be lost or eliminated in a cell type-specific manner. This loss can be achieved passively during DNA replication, or actively through enzymatic DNA demethylation. During active DNA demethylation, 5mC is oxidized to produce 5hmC, 5fC, and further 5caC in a replication-independent manner; subsequently, the DNA base-excision repair (BER) pathway can also remove the methylated cytosine by filling in an unmodified cytosine. In addition, passive elimination of 5mC is likely enhanced by active DNA demethylation, as DNMT1 (DNA methyltransferase 1) activity can be depleted by DNA with 5hmC *in vitro* (Valinluck and Sowers, 2007; Ji et al., 2014). More and more studies indicate that 5hmC acts not only as an intermediate during 5mC demethylation, but also plays important roles during maintenance of pluripotency in embryo stem cells (ESCs), neural system development and tumorigenesis (Thomson and Meehan, 2017). However, many questions remain to be answered. How DNA is actively demethylated? Where does 5hmC exist, and what roles does 5hmC have during development? These questions are the focus of this review.

## 5hmC, a Retrospective View

Shortly after the discovery of 5mC, the identification of 5hmC was published in *Nature* in 1952 (Wyatt and Cohen, 1952). Wyatt and Cohen (1953) showed that 5hmC exists as “a major constituent of the nucleic acid of a virus”, instead of cytosine or 5mC, in bacteriophages *T2, T4*, and *T6* of *Escherichia coli*. The 5hmC modification of the viral DNA makes it different from host DNA and thereby avoids degradation by the host (Wyatt and Cohen, 1953; Moréra et al., 1999). β-glucosyltransferase of bacteriophages can catalyze the transfer of glucose (Glc) from uridine diphosphoglucose (UDP-Glc) to 5hmC in double-stranded DNA (dsDNA), subsequently the glucosylation protects the infecting viral DNA from host restriction enzymes (Moréra et al., 1999). In 1972, 5hmC was also found in vertebrates, including rat, mouse, and frog (Penn et al., 1972), but 5hmC was only poorly understood as an epigenetic mark on DNA.

Further work on 5hmC had not attracted much attention until two important publications in *Science* in 2009 (Kriaucionis and Heintz, 2009; Tahiliani et al., 2009). Kriaucionis and Heintz (2009) reported that 5hmC comprises 0.6 and 0.2% nucleotides in Purkinje cells and granule cells, respectively; soon after 5hmC was detected in all tissues and cell types in mouse, with the highest levels (0.3–0.7%) found in the central nervous system (CNS) (Kriaucionis and Heintz, 2009; Globisch et al., 2010). Another breakthrough on 5hmC was the identification of TET1 (Ten-Eleven Translocation 1), an enzyme that oxidizes 5mC to 5hmC (Tahiliani et al., 2009). These new findings undoubtedly revitalized interest on 5hmC function, so much so that now 5hmC is accepted as the sixth DNA base in mammalian genomic DNA.

## How is 5hmC Produced and Eliminated?

### TET Proteins and 5hmC

Inspired by the biosynthesis of an unusual modified thymine J (β-D-glucosyl-hydroxymethyluracil) in Trypanosomes, Tahiliani et al. (2009) identified homologs of J-binding protein 1 (JBP1) and JBP2, two thymine (T) hydroxylases of the 2-oxoglutarate (2OG)- and Fe(II)-dependent oxygenase superfamily in mammals (Yu et al., 2007; Cliffe et al., 2009; Tahiliani et al., 2009). Human TET1, TET2, and TET3, as well as other orthologs from metazoa, fungi, and algae, were soon identified when databases were queried with the predicted oxygenase domains of JBP1 and JBP2 (Tahiliani et al., 2009). *TET1* was reported to fuse with the *MLL* gene to produce a chimeric transcript in acute myeloid leukemia (AML) (Ono et al., 2002). TET1 is directly responsible for the production and abundance of 5hmC. The oxidation of 5mC to 5hmC is executed by the predicted catalytic domain of TET1 (TET1-CD) in a Fe(II)- and 2OG-dependent manner (Tahiliani et al., 2009). Three TET proteins, TET1, TET2, and TET3, all exhibit oxidation activity to convert 5mC to 5hmC (Ito et al., 2010). In mouse embryonic stem cells (mESCs), the 5hmC level is decreased at the promoter/TSS (transcription start site) when TET1 is depleted, whereas TET2 is predominantly associated with 5hmC in gene bodies and at boundaries of actively expressed exons (Huang et al., 2014).

In addition, *TET* genes show cell/organ-specific expression. *TET1* is preferentially expressed in ESCs, while *TET2* and *TET3* exhibit similar expression profiles in various tissues (Ito et al., 2011; Nestor et al., 2012; Tollervey and Lunyak, 2012). *TET3* is abundantly expressed in the cerebellum, cortex, and hippocampus. TET1 and TET2 can catalyze the oxidation of 5mC and 5hmC in DNA (Gu et al., 2011; He Y.F. et al., 2011). In HEK293 cells transfected with *TET2*, 90% of the 5mC or 5hmC can be converted to 5caC. Using a two dimensional TLC (2D-TLC) assay, Ito et al. (2011) demonstrated that TET proteins have enzymatic activity able to oxidize 5mC to 5hmC, 5fC and 5caC *in vitro*. TET3 contributes to 5hmC production during gametogenesis and embryogenesis (Gu et al., 2011). Sperm and eggs possess distinct epigenomes that are subjected to reprogramming after fertilization. *TET3* is specifically expressed at the male pronuclear stage, and consistent with this, oxidation of 5mC occurs in the paternal genome and results in the accumulation of 5hmC (Gu et al., 2011; Amouroux et al., 2016). Furthermore, 5hmC generation in zygotes is also dependent on Dnmt3a and Dnmt1, two enzymes that produce 5mC. The fresh 5hmC comes from *de novo* oxidation of 5mC in zygotes (Amouroux et al., 2016).

Protein Lin28A regulates production of 5hmC by recruiting TET proteins during the conversion of 5mC to 5hmC. Lin28 was first discovered in *C. elegans* as an RNA binding protein. However, Zeng et al. (2016) reported that Lin28A, a paralog of Lin28 that is preferentially expressed in mouse embryos and ESCs before differentiation, can recruit TET1 to DNA and facilitate the conversion of 5mC to 5hmC and demethylation of gene bodies (Tan and Yeo, 2016).

### Excision of 5fC and 5caC

5hmC, 5fC, and 5caC show different abundances and tissue specificities. 5hmC is 10- to 100-fold more prevalent than 5fC/5caC and it is relatively enriched in neurons, stem cells, and much decreased in cancer cells (Tahiliani et al., 2009; Globisch et al., 2010; Ito et al., 2011; Song et al., 2011; Mellén et al., 2012). These differences are due to different preference and activity of TET proteins for these bases. Both human TET1 and TET2 show higher activity for 5mC than for 5hmC/5fC. Once 5hmC is established in genomic DNA, it is not easy to oxidize it to 5fC and 5caC (Hu et al., 2015). However, TET3 acts as 5caC reader during 5caC excision in BER, since its CXXC domain shows high affinity for 5caC (Jin et al., 2016).

**FIGURE 2:**
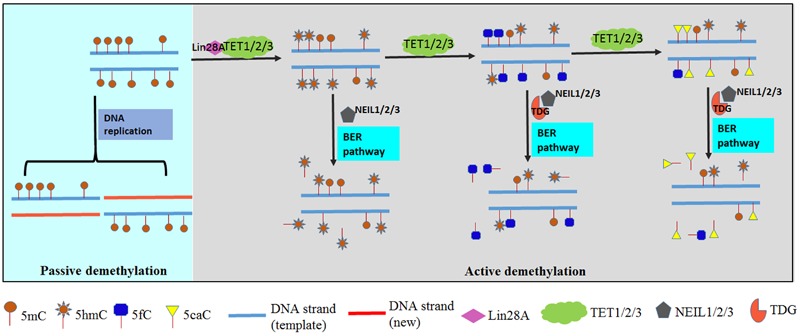
DNA demethylation in mammals. DNA demethylation is executed through passive demethylation (light blue chart) and active demethylation (gray chart). DNA methylation is maintained by DNA methyltransferases (DNMTs) during replication. 5mC will be replaced by C if methylation maintenance fails in passive demethylation. Active demethylation is achieved by Ten-Eleven Translocation (TET) proteins which can stepwise oxidize 5mC to 5hmC, 5fC and 5caC. Subsequently an abasic site is generated and filled in with an unmodified C by thymine-DNA glycosylase (TDG) and base-excision repair (BER) pathway. Furthermore, Lin28A is reported to recruit TET1 and NEIL proteins involved in excision of 5hmC, 5fC, and 5caC.

DNA repair proteins for DNA damage response play key roles in active DNA demethylation (Wossidlo et al., 2010; Spruijt et al., 2013). The distribution of 5fC can be regulated through TET-mediated oxidation and excision by thymine-DNA glycosylase (TDG). TDG is a DNA repair enzyme that excises T from G:T mispair and BER of deaminated 5mC or 5hmC. TDG is active on both 5fC and 5caC, but not 5mC or 5hmC (He Y.F. et al., 2011; Maiti and Drohat, 2011). The dynamic methylation status of 5mC is maintained by the antagonistic action of TET and TDG. On one hand, 5mC is stepwise oxidized by TET proteins to produce 5hmC, 5fC, and 5caC. On the other hand, TDG acts on 5fC and 5caC as a glycosylase to regenerate unmodified cytosine (C) through the BER pathway (**Figure 2**). This iterative oxidation is the main way active demethylation of 5mC is accomplished in mammals (**Figure 2**). Besides TDG, there are three orthologs of the prototypical endonuclease VIII (Nei). The Nei-like NEIL1-3 enzymes are alternative DNA glycosylases to TDG (Prakash et al., 2014). NEIL1, 2, and 3 can partially rescue the loss of TDG (Müller et al., 2014). Schomacher et al. (2016) reported that NEIL1 and NEIL2 promote TDG-mediated excision of 5fC and 5caC. It is likely that TDG first hydrolyses 5fC or 5caC, then TDG is displaced by NEIL1 and NEIL2, and subsequently the DNA backbone is cut in the BER pathway (Spruijt et al., 2013).

It is also speculated that 5hmC is alternatively deaminated by AID (Activation-induced deaminase) (Hendrich et al., 1999), to generate 5hmU and then to BER to complete 5mC demethylation. However, as AID cannot work on dsDNA, and the rate of deamination of 5hmC in single-stranded or dsDNA is also very low, the spontaneous deamination of 5hmC to 5hmU may not be able to compete with the oxidation step from 5hmC to 5fC. Furthermore, no detectable deamination of 5hmC has been found, even for cells overexpressing AID/APOBEC (Nabel et al., 2012). It is still not clear whether 5hmC can proceed with the deamination reaction. In this case, the development of sensitive methods for 5hmU detection and demonstration of 5hmC deamination in both *in vivo* and *in vitro* will be needed to answer the question (Grand et al., 2014; Lu et al., 2015).

## Where are 5hmC Bases?

### Genome Distribution of 5hmC and Gene Activity

5hmC is generated from 5mC by TETs, however, the TET protein level does not always reflect the existence of 5hmC. There may be post-transcriptional regulation or interaction partners of TETs, since 5hmC abundance is remarkably higher during neuronal development in mouse, even though there is no significant increase in *TET* family gene expression (Szulwach et al., 2011b).

First, to what extent can the TET proteins transfer 5mC to 5hmC? 5mC often occurs in pairs at CpG in reversely complemented DNA strands. Can both 5mCs be converted to two 5hmCs? Song et al. (2016) developed a technology, single-molecule fluorescence resonance energy transfer (smFRET), to image and quantify 5mC and 5hmC simultaneously (Branco et al., 2012). In this method, DNA is end-labeled with biotin and immobilized on a slide, then 5hmC and 5mC are labeled with Cy5 and Cy3 and act as donor (Cy5-5hmC) and receptor (Cy3-5mC), respectively. The hemihydroxymethylated/hemimethylated CpG sites (5hmC/5mCpGs) correspond to a high-FRET (∼0.78) state. By calculating the FRET, it was estimated that 5hmC/5mCpGs account for about 60% of 5hmCs (Song et al., 2016). This suggests that 5hmC, apart from CpG sites, also occurs in other parts of the genome.

5hmC is associated with gene transcription or translation. More 5hmC is found in gene bodies of active genes, and TET1 is often observed at TSS of genes with high CpG promoters that are marked by the bivalent histone signature of H3K27me3 and H3K4me3. It is therefore assumed that 5hmC and TET proteins may regulate gene expression through modulating chromatin accessibility of the transcriptional machinery, or by inhibiting repressor binding. This is consistent with 5hmC enrichment within gene bodies, promoters, and transcription factor (TF)-binding regions (Nestor et al., 2012). Immunostaining also showed that 5hmC often accumulated in gene-rich regions marked by H3K4me2/3 (Wion and Casadesus, 2006; Ficz et al., 2011; Szulwach et al., 2011a; Branco et al., 2012; Kubiura et al., 2012). The relationship between 5hmC peaks and gene expression level is complicated. For example, genes with active transcription show depleted 5hmC in their TSS regions, while genes of low expression show abundant 5hmC at promoters in both ESCs and NPCs (neural progenitor cells). However, in gene bodies, 5hmC peaks are positively correlated with gene expression levels in ESCs, but with lower expression levels in NPCs. Consistently, 5hmC distribution is vastly different in ESCs and NPCs. For example, the majority of 5hmC peaks of ESCs are lost in NPCs, yet some *de novo* DNA hydroxymethylation occurs at gene loci associated with mature neuronal functions (Tan et al., 2013). Apart from the different distribution of 5hmC on gene models, the global 5hmC level of NPCs is much lower than that of ESCs, which suggests that differentiation of ESCs to NPCs requires a genomic reduction of 5hmC (Tan et al., 2013). Consistently, *TET1* and *TET2* are highly expressed in mESCs, and 5hmC is enriched in mouse and human ESCs. Enrichment of 5hmC at the promoter/TSS is most likely associated with TET1, while the 5hmC level in gene bodies and boundaries of exons in active genes is correlated with TET2 activity (Huang et al., 2014).

Different studies about 5hmC abundance and gene expression are inconsistent. It seems that this is rather a cell type-dependent regulatory network than simple activation or repression of gene activity. Differentially hydroxymethylated regions (DHMRs) were also uncovered in mouse ESCs and NPCs (Tan et al., 2013). The tissue type is likely a main modifier for 5hmC, although 5hmC is accumulated in actively transcribed genes, for a gene in similar transcribed level, there may be 20-fold change of 5hmC on the gene body in different tissues (Nestor et al., 2012).

5hmC may be involved in regulating gene expression by association with various regulatory elements and processes (Szulwach et al., 2011a,b). Distribution of 5hmC is affected by histone modification, binding proteins of epigenetic marks, and chromatin configuration during cell differentiation and specification. 5hmC accumulated at the TSSs of genes whose promoters are decorated with dual histone marks, H3K27me3 for transcription repression, and H3K4me3 for transcription activation (Pastor et al., 2011), and at “poised” and active enhancers labeled with H3K4me1, H3K18ac, and H3K27ac (Szulwach et al., 2011a,b). Genes involved in development have “bivalent domains” (Bernstein et al., 2006) in promoters. In pluripotent ESCs, the “bivalent domains” can poise genes with both activating (H3K4me3) and repressive (H3K27me3) marks. The TET-mediated generation and distribution of 5hmC, global 5hmC/5mC change, and reprogramming of *de novo* “bivalent histone code” in CGI (CpG island) promoters contribute to bivalent domains directly (Hammoud et al., 2014; Grosser et al., 2015; Kong et al., 2016). TET1 forms a complex with Polycomb repressive complex 2 (PRC2) at H3K27me3 positive sections in the “bivalent” regions (Neri et al., 2013). 5hmC is abundant in both repressed (bivalent, TET1/PRC2-cobound) and activated (TET1-only) genes (Wu et al., 2011). This suggests that 5hmC plays a role in the machinery responsible for pluripotency switch.

The 5hmC level was changed in LINE (long tandem repeat), SINE (short interspersed nuclear element), and other repetitive elements in umbilical cord DNA from gestational diabetes mellitus (GDM) or preeclampsia patients (Sun et al., 2016). It is unclear whether 5hmC plays a role in regulating transposon activity.

As a reader for both 5mC and 5hmC (Mellén et al., 2012; Spruijt et al., 2013; Diotel et al., 2017), MeCP2 (methyl-CpG-binding protein 2) has similar affinity to 5hmC and 5mC (Mellén et al., 2012). However, the 5hmC level is negatively correlated with MeCP2 abundance. The binding of MeCP2 with 5mC may possibly hinder the production of 5hmC (Szulwach et al., 2011a). 5hmC accumulates in MeCP2- and H3K4me2-enriched nuclear foci. Thus 5hmC and MeCP2 might constitute a cell-specific epigenetic mechanism for the regulation of chromatin structure and gene expression.

### Cell Type-Specific and Developmentally Related Abundance of 5hmC

Global detection of 5hmC has been carried out in human, mouse, zebrafish, and Xenopus (Globisch et al., 2010; Munzel et al., 2010; Chen and Riggs, 2011; Jin et al., 2011; Song et al., 2011; Szulwach et al., 2011a,b; Almeida et al., 2012; Han et al., 2016; Diotel et al., 2017). These studies showed that the dynamics and abundance of 5hmC are cell type-dependent and developmentally regulated. It is interesting that in amphibians, high levels of 5hmC were detected in Xenopus spinal cord and axolotl neural tube cells, or distributed in amphibian skin and connective tissue in a mosaic manner (Almeida et al., 2012). Significant increases of Sertoli cell-specific global 5hmC were detected during rat puberty. From juvenile to adult rat, the functions of Sertoli cells are different, and genes which lose or gain 5hmC belong to different functional pathways (Landfors et al., 2017).

Recently, Yu et al. (2012) adapted traditional bisulfite sequencing to map 5hmC globally. TET-Assisted Bisulfite Sequencing (TAB-Seq) showed that 5hmC was 10-fold more abundant in CNS and ESCs than in peripheral tissues (Branco et al., 2012). The distribution of 5hmC is somewhat negatively correlated with 5mC along genomic DNA in ESCs. Its frequency is biased with DNA sequence and strand asymmetry in a tissue-dependent manner. In mouse, for example, 5hmC is developmentally enriched and age-dependent in brain. Furthermore, genes that have acquired 5hmC during aging are associated with age-related neurodegenerative disorders (Song et al., 2011; Szulwach et al., 2011a). 5hmC acquisition occurs in developmentally programmed neuronal cells, since very low or no 5hmC was detected in the immature neurons. However, a significant increase of 5hmC was found from postnatal day 7 (P7) to adult in both cerebellum and hippocampus (Szulwach et al., 2011a). This leads to the establishment of DhMRs and the transcriptional programing of tissue-dependent genes in cerebellum and hippocampus (Yao et al., 2012).

Recently, a nano-hmC Seal method (Song et al., 2012; Han et al., 2016) enabled genome profiling of 5hmC from DNA isolated from 1000 cells. The 5hmC pattern was associated with hematopoiesis, as the enhancer sites were dynamically hydroxymethylated during hematopoietic stem cell (HSC) differentiation. Chromatin Immunoprecipitation sequencing (ChIP-seq) and Assay for Transposase-Accessible Chromatin sequencing (ATACT-seq) were used to show that the 5hmC density in gene bodies correlates positively with histone modification marks of gene activation, such as H3K4me1 and H3K4me2, in all cell types during hematopoiesis. However, 5hmC and H3K4me1 were not concentrated in the center of ATACT-seq peaks with high chromatin accessibility, but instead were enriched in ATACT-seq peaks with lower signal intensity or the less active chromatin elements (Chen and Riggs, 2011).

Furthermore, 5hmC modification is associated with tumorigenesis and stress response. Compared to healthy tissues, the abundance of 5hmC is reduced up to eightfold in cancer cells (Jin et al., 2011; Lian et al., 2012). TET2 depletion led not only to the loss, but also the redistribution of 5hmC in *tet2*-mutant AML murine stem cells (Han et al., 2016). Potential stress-related target genes were proposed to be regulated by 5hmC in early-life stressed mouse (Papale et al., 2017). The glucocorticoid receptor Nr3C1 in the hippocampus is highly mediated in stress response. 5hmC is increased about 1.8-fold at the seven CpGs in the 3′ UTR of *Nr3C1* gene after 30 min restraint in a conical vial and 1 h recovery (Li et al., 2015). In total, 458 hyper- and 174 hypo-DhMRs were identified in hippocampal DNA, including 470 and 166 genes that have hyper- and hypo-DhMRs, respectively, and 2 genes that showed both hyper- and hypo-DhMRs. Among them, more than 1/3 of the genes (240 in 638) are involved in stress responses. All the functional DhMRs contain at least one TF binding motif. These results suggest that 5hmC possibly acts through TFs, in that the affinity of TFs to DNA is changed in accordance with 5hmC modification (Li et al., 2016).

Many studies showed that 5hmC is linked with neurological and psychiatric disorders. Rett syndrome (Szulwach et al., 2011a), aging and Alzheimer’s disease (Chouliaras et al., 2013; Shu et al., 2016; López et al., 2017), melanoma (Papale et al., 2017), Huntington’s disease (Villar-Menéndez et al., 2013; Wang et al., 2013), Fragile X-associated tremor/ataxia syndrome (FXTAS) (Yao et al., 2014), Ataxia-telangiectasia (Jiang et al., 2015), Schizophrenia, bipolar disorder, major depressive disorder (Dong et al., 2012; Matrisciano et al., 2013; Tseng et al., 2014), Autism (Penagarikano et al., 2011), and intracerebral hemorrhage (Tang et al., 2016), all feature obvious changes of 5hmC. Aberrant life will be caused when the dynamics and distribution of 5hmC, or even the ratio of 5hmC to 5mC, are disturbed. It is widely accepted that when the abundance and distribution of 5hmC change, disease occurs.

## What are the Mechanism and Function of 5hmC?

Rediscovery of 5hmC in DNA inspired studies on the distribution and function of 5hmC. 5hmC modification of the phage DNA prevents restriction and degradation during infection into *E. coli* host (Hattman and Fukasawa, 1963; Shedlovsky and Brenner, 1963; Erikson and Szybalski, 1964). Some new enzymes are produced in the bacterium infected by phage with 5hmC modified DNA (Flaks and Cohen, 1959; Kornberg et al., 1959; Koerner et al., 1960). Substitution of C for 5hmC in vegetative *T4* DNA depresses the synthesis of late proteins (Hosoda and Levinthal, 1968; Kutter and Wiberg, 1969), moreover, transcription of some late genes can only occur from 5hmC-containing, but not from C-containing DNA (Kutter and Wiberg, 1969).

The function and mechanism of 5hmC are still elusive. Distribution of 5hmC is diversified at enhancer, promoter, TSS, gene body, 3′ UTR or intragenic region. It is conceivable that 5hmC takes roles as *cis* element, for example, to promote or repress gene expression by binding to TFs, such as activators or repressors in regulatory regions of a gene.

The second way for 5hmC to work as *cis* player is to associate with histone modifications for the alteration of chromatin configuration, to switch “on” or “off” genes in heterochromatic or euchromatic chromatin, respectively. As mentioned above, TET proteins, 5hmC, 5mC and histone modifications such as H3K27me3 and H3K4me3, combine together to form the complicated bivalent domains of poised genes to maintain pluripotency of ESCs.

The third way in which 5hmC functions is possibly to modulate alternative splicing facilitated by 5hmC distribution in gene bodies, and exon/intron boundaries. Alternative splicing is a conserved way to diversify the transcriptome and proteome of a given genome. Transcripts from about 90% genes undergo alternative splicing (Wang et al., 2008). About 30 years ago it was shown that, splicing can occur co-transcriptionally in *Drosophila* (Beyer and Osheim, 1988). RNA polymerase II (Pol II) can recruit the 5′ cap-binding complex (CAP), splicing and pre-spliceosome factors, and the polyadenylation complex in the context of nucleosome-containing chromatin (Luco et al., 2011). DNA modification and alternative splicing are linked by protein bridge of a variety of components such as CCCTC-binding factor (CTCF), MeCP2, and HP1 (Heterochromatin Protein 1) (Iannone and Valcarcel, 2013; Maunakea et al., 2013; Lev Maor et al., 2015; Yearim et al., 2015). 5hmC is more abundant in constitutive exons than in alternatively spliced exons (Khare et al., 2012). It is likely that 5hmC, similar to 5mC, plays a role in alternative splicing through these regulatory proteins. DNA modification contributes to binding of these proteins to DNA. Pol II elongation, recognition of alternative exons by spliceosome, and the subsequent inclusion or exclusion of exons are promoted or paused by DNA methylation (Lev Maor et al., 2015). Rett syndrome is caused by the inhibition of binding of MeCP2 to 5hmC (Mellén et al., 2012), additionally, an increase of 5hmC and decrease of CTCF occupancy at the *FXN* 5′ UTR were also reported in Friedreich ataxia (Al-Mahdawi et al., 2013).

In addition, 5hmC is possibly linked with small RNA pathways. MiR29b, a key member of miR-29 family, is upregulated during differentiation of mESCs. MiR29b targets the 3′ UTR of *TET1*/*TET2* mRNAs, however, 5hmC level is decreased by repressing of *TET1*, but not *TET2* (Tu et al., 2015). The miR29b-TET1 axis promotes formation of mesendoderm lineage by inducing the Nodal signaling pathway and expression of involved genes. MiR29b may associate with active DNA demethylation by repressing TDG. Taken together, 5hmC are closely linked with miRNA regulation in formation of mesendoderm lineage (Tu et al., 2015).

## 5hmC Modification and Active DNA Demethylation in Plants

In plants, 5mC is often found in contexts of CG, CHG, and CHH (H represents either A, C or T). The *de novo* DNA methylation and maintenance are associated with different pathways. In *Arabidopsis*, Domains Rearranged Methyl-transferase 2 (DRM2), DNA Methyl-Transferase 1 (MET1), Chromomethylase 3 (CMT3), CMT2 and DRM2 are responsible to maintain DNA methylation in different pathways or sequence contexts. DNA demethylation also occurs passively or positively in plants. However, TET proteins or UHRF2 (Ubiquitin-like with PHD and Ring Finger Domains 2) (Spruijt et al., 2013; Zhou et al., 2014), the writers or readers of 5hmC found in mammals, have not been identified in plants, thus DNA demethylation might not be processed through TET pathway. Active DNA demethylation by BER pathway is initiated by ROS1 (Repressor of Silencing 1) and other DME family members, DME, DML2, and DML3, the bifunctional DNA glycosylase and apyrimidinic (AP) lyase (Gehring et al., 2006; Morales-Ruiz et al., 2006). The DME glycosylases can remove 5mC from the DNA backbone directly and create an abasic site subject to the BER pathway (Gong and Zhu, 2011). However, mutations in the known demethylases in *Arabidopsis* do not cause global demethylation, but only affect the methylation status of some specific loci (Gehring et al., 2006; Lister et al., 2008). It is still elusive that how global demethylation occurs during gametogenesis and embryogenesis in plants. The BER pathway may not be the main pathway for global demethylation, because it will result in too many abasic sites and broken strands simultaneously, which could destabilize the whole genome (Zhu, 2009). Does this mean that any other pathway is involved in active DNA demethylation in plants? Is the unknown active demethylation pathway in plant similar to the TET pathway in mammals?

Repressor of Silencing 1 and DME purified from *E. coli* can excise 35-mer oligonucleotides containing cytosine, 5mC, 5hmC, 5fC, and 5caC, indicating that they are able to cleave both 5mC and 5hmC *in vitro*. However, 5hmC was not detected in the *Arabidopsis* genome (Jang et al., 2014). Several other groups also tried to detect if there is 5hmC in plants. Up to now, the existence of 5hmC in plants is still under debate (Terragni et al., 2012; Yao et al., 2012; Liu et al., 2013; Moricová et al., 2013; Wang et al., 2015). Wang et al. studied three rice cultivars and concluded that 5hmC modification is present in rice DNA, although in low abundance as 1.39 ± 0.16 and 2.17 ± 0.03 per million nucleosides in leaf and panicle, respectively. 5hmC distribution tends to localize in TE and heterochromatin regions in rice (Wang et al., 2015). Based on the extremely low abundance of 5hmC, the lack of genes encoding proteins for the generation, recognition, and functioning of 5hmC, and activity of ROS1 and DMEs to eliminate 5mC efficiently and directly, it is assumed that 5hmC, if any, may be an intermediate of passive demethylation in local regions, or not an enzymatic product in plants.

## Perspective: More Questions are Waiting

Benefiting from the development and progress of sequencing technology, biochemistry techniques, and cellular and molecular strategies, epigenetic research has been blooming in the past 20 years. It is no doubt that the content of epigenetic research has been enriched from studies on 5hmC and other recently determined epigenetic marks, such as methylated adenine in DNA, or even in RNA. It is suggested that 5hmC most likely plays a role in gene expression, pluripotency of stem cells, stress response, disease and aging. However, functional studies on 5hmC are still ahead. There are still some fundamental questions to be addressed:

(1) There is a long journey from global detection of 5hmC modification to precise elucidation of functional genes, pathways and regulatory mechanisms.

(2) More proteins that produce, recognize, and eliminate 5hmC must be identified. It is badly needed to uncover the mechanism underlying 5hmC function. The functional studies will be aided, in turn, by research on 5hmC writers, readers, and erasers.

(3) Does 5hmC exist in mitochondrial DNA (mtDNA)? Given that 5hmC is present in mtDNA, does it share the similar pattern with 5hmC in nuclear DNA? Is 5hmC involved in retrograde signal transduction from organelle to nucleus? And is there any evolutionary relation between 5hmC in mtDNA with that in bacteriophage DNA?

(4) RNA hydroxymethylcytosine (hmrC or hm5C, for the difference of 5hmC in DNA), but not 5mC, or 5hmC in DNA was reported in *Drosophila* (Delatte et al., 2016). Does it mean any preference of DNA or RNA modification in some organisms? Do the proteins which are involved in DNA epigenetic modification act in similar way in RNA?

(5) Since the distribution and abundance of 5hmC in DNA correlate with some mental or neuronal disorders, is the change of 5hmC a cause or a result of the diseases? Will the diseases be cured or prevented when 5hmC distribution alteration is prohibited by some gene therapy, if it is a cause of the disorders?

(6) Is it possible that 5hmC is enriched in some special cells or at appropriate developmental stages, but cannot be detected globally in plants? Can we obtain 5hmC genomic DNA if we introduce and express *TET* genes *in planta*? What will happen if 5hmC modification occurs in plant DNA?

## Author Contributions

D-QS and W-CY conceived the review. D-QS prepared manuscript. IA, JT, and W-CY discussed the proposal and revised manuscript.

## Conflict of Interest Statement

The authors declare that the research was conducted in the absence of any commercial or financial relationships that could be construed as a potential conflict of interest.
